# Rate of Change in Bruch’s Membrane Opening-Minimum Rim Width and Peripapillary RNFL in Early Normal Tension Glaucoma

**DOI:** 10.3390/jcm9082321

**Published:** 2020-07-22

**Authors:** Hyun-kyung Cho, Changwon Kee

**Affiliations:** 1 Department of Ophthalmology, Gyeongsang National University Changwon Hospital, Gyeongsang National University School of Medicine, Changwon 51472, Korea; 2 lnstitute of Health Sciences, School of Medicine, Gyeongsang National University, Jinju 52727, Korea; 3 Department of Ophthalmology, Samsung Medical Center, Sungkyunkwan University School of Medicine, Seoul 06351, Korea; ckee@skku.edu

**Keywords:** Bruch’s membrane opening minimum rim width, early normal tension glaucoma, normal tension glaucoma, glaucoma progression, optical coherence tomography, retinal nerve fiber layer

## Abstract

Background: to investigate the rate of change (ROC) of Bruch’s membrane opening minimum rim width (BMO-MRW) and peripapillary retinal nerve fiber layer (RNFL) thickness in early normal tension glaucoma (NTG) patients. Methods: in this longitudinal cohort study, 115 subjects (115 eyes) diagnosed as early NTG (mean deviation > −6.0 dB) and who had completed more than five times of spectral-domain optical coherence tomography (OCT) tests with acceptable quality were included. Measurement of BMO-MRW and RNFL were performed at 3-month intervals by OCT. Linear mixed-effects model was employed to calculate the ROC in global region and six Garway-Heath sectors with adjusting age, sex, and BMO area. Results: Average follow-up was 20.99 ± 6.99 months with OCT number of 7.54 ± 2.12. Baseline intraocular pressure was 14.72 ± 2.70 mmHg and MD was −2.73 ± 2.26 dB. ROC of global BMO-MRW was −2.06 ± 0.65 µm/yr and RNFL was −0.96 ± 0.16 µm/yr (*p* = 0.098). The most rapid ROC was in inferotemporal sector (BMO-MRW: −3.02 ± 0.88 µm/yr, RNFL: −1.96 ± 0.36 µm/yr) followed by superotemporal sector. Conclusion: The ROC of BMO-MRW, the new parameter along with that of RNFL should be considered in the management of early NTG. BMO-MRW may show visible reduction ROC better than RNFL to detect early progression in early NTG when visual field may not show significant change.

## 1. Introduction

Glaucoma is a progressive optic neuropathy accompanied by a deficit of the retinal nerve fiber layer (RNFL) with a corresponding thinning of neuroretinal rim (NRR) tissue that can lead to progressive visual field defect [1,2]. Noticeable structural changes by current instruments may precede functional visual field loss in an early stage of glaucoma progression [3,4,5]. In this regard, parameters of the structure acquired by optical coherence tomography (OCT) might be more advantageous in identifying initial progressive changes than a standard automated perimetry.

Nowadays, a relatively new parameter, Bruch’s membrane opening-minimum rim width (BMO-MRW), has been introduced in the assessment of optic discs [6,7,8,9,10]. BMO-MRW measures the shortest length between BMO and internal limiting membrane (Figure 3D). It provides consistent borders of optic nerve head with more precise estimation of the NRR than conventional ophthalmic inspections [6,7,8,11]. It has been lately shown that BMO-MRW provides better performance in the diagnosis of glaucoma than traditional NRR parameters [8,9,10]. BMO-MRW also shows a better structure-function relationship in glaucoma than other pre-existing peripapillary RNFL and parameters of confocal scanning laser ophthalmoscope [10,11,12,13]. We have previously described a disagreement between BMO-MRW and RNFL in classification analysis of color-codes [14]. At early glaucomatous stage, structural changes are minimal. In addition, different structural parameters might display inconsistent outcomes. Thus, one structural parameter may have more advantage than the other parameter in a specific situation.

Normal tension glaucoma (NTG) is more prevalent in Asians than in other races. NTG accounts for the majority (mean of 76.3%) of open angle glaucoma in Asians [15]. However, the rate of change of structural parameters of NTG, especially BMO-MRW, a new parameter, has not been studied well before, particularly not in early NTG. Considering that a change of structural parameter is more important in early NTG than in advanced stage, investigating the rate of change (ROC) of OCT-based parameters, for instance, RNFL and BMO-MRW, would have significant meaning. Longitudinal ROC or progression rate is important in determining treatment intensity and follow-up period in the management of glaucoma. Nevertheless, detecting glaucomatous progression is challenging in clinical practice, particularly in the case of NTG. Ethnicity and glaucoma subtype predominance might have an important influence on longitudinal ROC [16].

The ROC of RNFL in Asian (Korean) cohorts including predominantly NTG with early stage (baseline mean deviation, MD: −2.48 ± 4.46 dB) has been shown to be −0.71 (−0.94; −0.48) µm/yr [16]. In another study on moderate stage of NTG (baseline MD: −8.49 ± 7.63 dB in progressed group, −8.13 ± 9.58 dB in non-progressed group) in Asian (Korean) subjects, the ROC of RNFL was −0.39 ± 0.24 µm/yr in progressed group and −0.07 ± 0.29 µm/yr in non-progressed group [17]. Considering the relatively small ROC of RNFL, which is less than −1.0 µm/yr in Asian cohorts with NTG predominance, early detection of glaucomatous deterioration with subtle change of RNFL in clinical situation may be difficult.

The aim of this longitudinal cohort study was to investigate ROCs of BMO-MRW and RNFL in patients with early NTG. ROCs were calculated for these two structural parameters in a single ethnic group of Asians with early NTG. We intended to identify which structural parameter would be more useful in the detection of early glaucomatous progressive change in patients with early stage of NTG.

## 2. Materials and Methods

This retrospective, observational, cohort study was conducted in accordance with the tenets of the Declaration of Helsinki. The present study was approved by the Institutional Review Board of Gyeongsang National University Changwon Hospital, Gyeongsang National University, School of Medicine (GNUCH-2019-09-031-001). The requirement for informed consent was exempted by the IRB because this was a retrospective study.

### 2.1. Subjects

Subjects were evaluated in the glaucoma clinic at Gyeongsang National University Changwon Hospital by a single glaucoma specialist (H.-k.C.). Among 285 subjects diagnosed as NTG, those who qualified the inclusion criteria shown below were included in the final analysis.

NTG was defined as the following: an IOP of ≤21 mmHg without treatment and findings of glaucomatous optic disc injury and corresponding VF defects, an open angle inspected by gonioscopy, and no other cause of optic disc impairment than glaucoma [18]. Early NTG was defined if VF test result of mean deviation (MD) was more than −6.0 dB. Visual field test had to qualify the following reliability criteria: fixation loss of <20%; a false positive rate of <15%; and a false negative rate of <15%.

All subjects went through standard ophthalmic evaluations, including Spectralis spectral-domain OCT (Glaucoma Module Premium Edition, Heidelberg Engineering, Germany) and standard automated perimetry (HFA model 840; Humphrey Instruments, Inc., San Leandro, CA, USA). BMO-MRW and RNFL were measured at 3-month intervals with spectral-domain OCT. Those who had had acceptable quality of both BMO-MRW and RNFL measurements for more than five times were included. When both eyes met the inclusion criteria, only one eye was randomly selected.

Criteria for exclusion were as the following: poor images owing to eyelid blinking or poor fixation, any history of intraocular surgery but for uneventful phacoemulsification, any history of other optic neuropathies except for glaucoma (e.g., optic neuritis, acute ischemic optic neuritis), history of an acute angle-closure crisis that could affect the thickness of the BMO-MRW or RNFL, and any retinal disease that led to retinal swelling or edema and succeeding swelling of BMO-MRW or RNFL. A total of 115 subjects (115 eyes) with early NTG were included in the final analysis.

### 2.2. Optical Coherence Tomography

Images of optic disc were acquired with a Spectral-Domain OCT (Heidelberg Engineering, Germany) engaging the Glaucoma Module Premium Edition by a skilled technician. Twenty-four radial B-scans were obtained for BMO-MRW. A scan circle diameter of 3.5 mm among three scan circle diameters (diameters of 3.5, 4.1, and 4.7 mm) was selected for peripapillary RNFL thickness. Images with accurate centration and precise segmentation of the retina and quality scores of more than 20 were used for this study. OCT images were analyzed in an individual specific axis (FoBMO axis), which was the axis between the BMO center and the fovea of macula. Applying this FoBMO axis enabled more accurate analysis of each sector regarding individual cyclotorsion and more accurate comparison analysis with normative database than the conventional way of applying mere clock-hour positions [19].

### 2.3. Statistical Analysis

To determine the progression rate, or ROC as a regression coefficient which was the slope of each parameter (BMO-MRW and RNFL), we used a generalized linear mixed-effects model including a random intercept. ROCs in the global region and in each Garway-Heath sector were calculated with the linear mixed-effects model after adjusting for age, sex, and BMO area. Comparative analyses of the ROC of BMO-MRW and RNFL values in the global region and each sector were performed with a *t*-test. Because scales of BMO-MRW and RNFL were different, alike baseline values, percent reduction rates of coefficients along with standardized coefficients were compared. Percent coefficient was calculated by setting the initial intercept to 100 at time 0. Standardization was conducted by setting the mean as 0.0 and standard deviation as 1.0. *p* values of <0.05 were considered statistically significant. All statistical analyses were executed using SAS software version 9.4 (SAS Institute, Cary, NC, USA).

## 3. Results

### 3.1. Baseline Characteristics

Of 285 subjects, 115 subjects (115 eyes) with early NTG were included in the final analysis. The mean follow-up period was 20.99 ± 6.99 months. The mean number of OCT tests was 7.54 ± 2.12. The mean age of included subjects was 56.08 ± 10.94 years. Among these subjects, 53 (46.09%) were women. Eleven (9.57%) subjects had a family history of glaucoma. Optic disc hemorrhage was observed in 35 (30.43%) of 115 subjects.

The mean spherical equivalent (SE) was −1.69 ± 2.81 Diopters from all subjects. The mean baseline IOP was 14.72 ± 2.70 mmHg. Average central corneal thickness (CCT) was 540.13 ± 36.76 µm. The visual field index (VFI) was 93.30 ± 6.40% with a mean deviation (MD) and a pattern standard deviation (PSD) of −2.73 ± 2.26 dB and 4.75 ± 3.02 dB, respectively (Table 1). The mean BMO-fovea angle was −6.16 ± 3.17°. Quality scores of RNFL and BMO-MRW were fairly good (mean scores of 29.44 ± 3.53 and 32.38 ± 3.13, respectively, Table 1).

### 3.2. Baseline BMO-MRW and RNFL and the Rate of Change for Each Parameter

Baseline BMO area was 2.29 ± 0.54 mm^2^ (Table 2). Baseline BMO-MRW and RNFL of global region were 202.12 ± 44.47 µm and 79.29 ± 12.65 µm, respectively (Table 2). Baseline values of BMO-MRW and RNFL for six Garway-Heath sectors are shown in Table 2.

ROCs of BMO-MRW and RNFL for the global region were −2.063 ± 0.649 µm/yr and −0.956 ± 0.160 µm/yr, respectively (Table 2). ROCs of BMO-MRW and RNFL for the inferotemporal sector were −3.024 ± 0.882 µm/yr and −1.964 ± 0.363 µm/yr, respectively. ROCs of BMO-MRW and RNFL for the superotemporal sector were −2.401 ± 0.775 µm/yr and −1.250 ± 0.309 µm/yr, respectively. ROCs of BMO-MRW and RNFL for other sectors are shown in Table 2.

### 3.3. Comparison of the Rate of Change between BMO-MRW and RNFL

**Figure 1:**
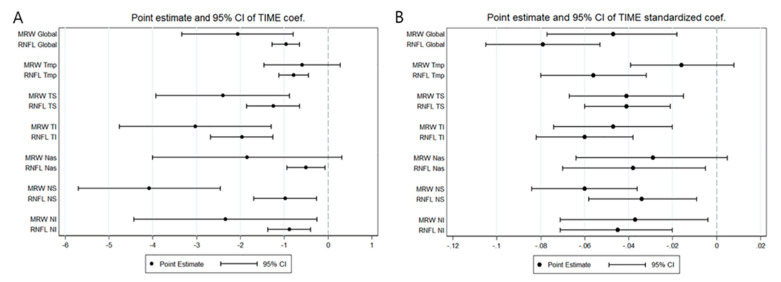
Confidence interval of each rate of change for BMO-MRW and RNFL from the global region and six Garway-Heath sectors. (**A**) The 95% confidence interval (CI) of the regression coefficients or progression rates in BMO-MRW and RNFL from the global region and the six Garway-Heath sectors, which overlap with each other except for the superonasal sector, suggesting that each value of BMO-MRW and RNFL was not significantly different in the global region and five sectors. (**B**) The 95% CI of the “standardized” regression coefficients or progression rates for BMO-MRW and RNFL from the global region and all six Garway-Heath sectors show overlap with each other, implying that these two standardized parameters are not significantly different in all sectors and the global region. The rates of change were additionally compared after standardization because BMO-MRW and RNFL differ in baseline values and scales. BMO-MRW: Bruch’s Membrane Opening-Minimum Rim Width, RNFL: retinal nerve fiber layer, Tmp: temporal, TS: superotemporal, TI: inferotemporal, Nas: nasal, NS: superonasal, NI: inferonasal.

**Figure 2:**
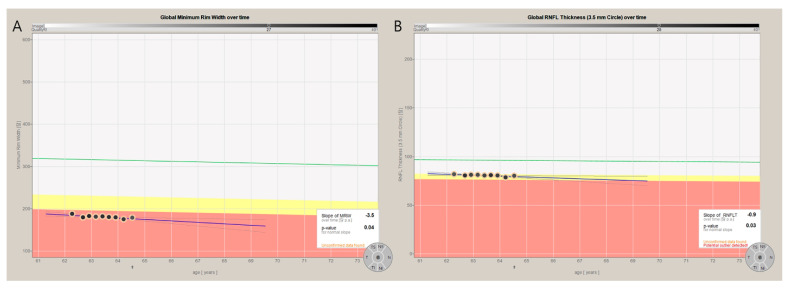
Representative case showing rate of change from the global region in BMO-MRW and RNFL. A representative case demonstrating the slope of BMO-MRW and RNFL of early NTG from November 2017 to February 2020 is shown. A 61-year-old female with the baseline MD of −4.19 dB and SE of −0.38 D is presented. Simple regression analysis estimating the slope of merely the global BMO-MRW (**A**) and the RNFL (**B**) by automated installed software of OCT is displayed. Note that the slope of BMO-MRW is −3.5 µm/yr, while the slope of the RNFL is −0.9 µm/yr. The *p*-values of both slopes obtained from nine times of OCT tests were significant (*p* = 0.04 and *p* = 0.03, respectively). BMO-MRW: Bruch’s Membrane Opening-Minimum Rim Width, RNFL: retinal nerve fiber layer.

ROC per year did not show significant difference between BMO-MRW and RNFL for the global region for five Garway-Heath sectors (temporal, superotemporal, inferotemporal, nasal, and inferonasal) (*t*-test, all *p* > 0.05). However, it did show significant difference for the superonasal sector (*t*-test, *p* = 0.0006) (Table 3). The 95% confidence intervals (CIs) of regression coefficients or ROCs in BMO-MRW and RNFL for the global region and five Garway-Heath sectors did overlap with each other, suggesting that ROC of BMO-MRW was not significantly different from that of RNFL. However, 95% CIs of coefficients or ROC in BMO-MRW and RNFL from superonasal sectors did not overlap with each other, including point estimates. This implied that ROCs of these two parameters for the superonasal sector were significantly different (Figure 1A). These findings of Figure 2A were consistent with findings of ROC comparison analysis shown in Table 3.

Although ROCs between BMO-MRW and RNFL were not significantly different for global region and almost all sectors, the ROC of BMO-MRW was consistently greater than that of RNFL in the same region and sectors (except for the temporal sector) in early NTG.

**Figure 3:**
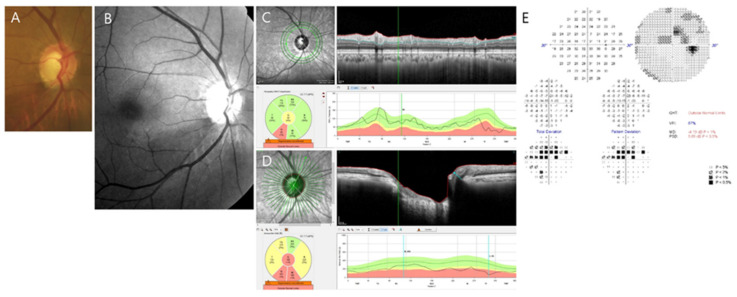
Initial presentation of the representative case. (**A**) Disc photography of the right optic disc of a 61-year old female with baseline intraocular pressure of 15 mmHg and central corneal thickness of 566 µm. Note the enlarged cupping and neuroretinal rim thinning of the inferotemporal area. (**B**) Red-free fundus photography showing corresponding inferotemporal retinal nerve fiber layer (RNFL) defect. (**C**) Optical coherence tomography (OCT) image of RNFL showing RNFL defect at the same inferotemporal sector. (**D**) OCT image of Bruch’s Membrane Opening-Minimum Rim Width (BMO-MRW) showing definite neuroretinal rim thinning at inferotemporal sector from BMO-based optic disc margin. BMO-MRW measures the minimal length between BMO and internal limiting membrane (red line). BMO-MRW of global region and six Garway-Heath sectors are displayed. The FoBMO axis is the axis between the fovea of the macula and the center of BMO. The FoBMO was employed for sectoral analysis, which enables more precise sectoral analysis because the cyclotorsion of individual eye is adjusted and considered for analysis. (**E**) Humphrey visual field at the initial presentation. Superior paracentral scotomas corresponding to the inferotemporal RNFL defect are noted. Baseline mean deviation was −4.19 dB, indicating early stage of NTG.

A representative case demonstrating the slope of BMO-MRW and RNFL of early NTG from November 2017 to February 2020 is shown in Figure 2. A 61-year-old female with baseline MD of −4.19 dB (Figure 3) and SE of −0.38 D is presented. Simple regression analysis was used to estimate the slope of BMO-MRW (Figure 2A) and RNFL (Figure 2B) for the global region using an automated installed software of OCT. Note that the slope of BMO-MRW was −3.5 µm/yr, while the slope of RNFL was −0.9 µm/yr. *p*-values of both slopes obtained from nine times of OCT tests were significant (*p* = 0.04 and *p* = 0.03, respectively).

### 3.4. Comparison of the Rate of Change in Percent Reduction between BMO-MRW and RNFL

As baseline values of BMO-MRW and RNFL were different and scales of these two parameters were also different, ROCs or progression rates in percent reduction were also compared. The progression rate calculated in percent reduction did not show significant difference between BMO-MRW and RNFL for the global region or the five Garway-Heath sectors except for the superonasal (SN) sector (all *p* > 0.05 except *p* = 0.0294 for SN) (Table 4).

### 3.5. Comparison of Rate of Change in BMO-MRW and RNFL after Standardization

ROCs or progression rates were also compared between BMO-MRW and RNFL after standardization because these two parameters had different baseline values and scales. Standardized regression coefficients or progression rates of BMO-MRW and the RNFL for the global region were −0.047 µm/yr (95% CI: −0.077–−0.018) and −0.079 µm/yr (95% CI: −0.105–−0.053), respectively. Other standardized coefficients or progression rate are summarized in Table 5.

The 95% CI of the standardized regression coefficient or progression rate of BMO-MRW and the RNFL for the global region or each Garway-Heath sector did not show an overlap with each other, suggesting that these standardized coefficients of the two parameters were not significantly different in any Garway-Heath sector or the global region (Figure 1B).

## 4. Discussion

To the best of our knowledge, the present study is the first to investigate the ROC of BMO-MRW in early NTG in a single ethnic group of Asians. The ROC of BMO-MRW was consistently greater than that of RNFL in the global region and each corresponding sector except for the temporal sector in early NTG. Although there were no statistically significant differences in ROCs between BMO-MRW and RNFL for any sector except for the superonasal sector, this result might be due to the relatively slow ROC of early NTG. Considering that the ROC of RNFL is relatively slow at less than 1.0 µm/yr, the ROC of BMO-MRW might be more beneficial than RNFL to detect significant change of glaucomatous deterioration earlier because BMO-MRW shows visible reduction rate of change better than RNFL. Identifiable structural changes precede functional visual field loss in the early stage of glaucoma progression [3,4,5]. Thus, structural parameters acquired by OCT might be more advantageous in detecting early progressive changes than a standard visual field test.

NTG mainly (76.3%) consists of open-angle glaucoma in Asian population. This has been described in a review article of population-based glaucoma-prevalence studies in Asians [15]. The proportion of NTG among primary open angle glaucoma was 77.1% (2.7% among 3.5%) in the Namil study, a population based glaucoma study conducted in central South Korea [20]. In a previous study that calculated the ROC of RNFL in Asian (Korean) cohorts that included predominantly NTG, the ROC of RNFL was −0.71 (−0.94; −0.48) µm/yr in early stage of glaucoma (baseline MD: −2.48 ± 4.46 dB) [16]. In a recent study of Korean subjects, the reduction rate of RNFL from early NTG cluster group was −0.83 µm/yr with baseline MD of −3.19 ± 4.13 dB [21]. Therefore, it might not be so easy to see a change of glaucomatous progression with RNFL, which was less than −1.0 µm/yr in the early stage of NTG. However, the ROC of BMO-MRW as a new parameter in NTG patients, especially for those with early NTG, has not been reported before.

The ROC in the present study for those with early NTG was −0.956 ± 0.160 µm/yr for global RNFL and −2.063 ± 0.649 µm/yr for global BMO-MRW. The ROC of RNFL showed similar rate with those reported in previous studies [16,21], which was also less than −1.0 µm/yr. However, the ROC of BMO-MRW, a new parameter, showed much greater rate than that of RNFL.

Baseline values of BMO-MRW and RNFL thickness can influence each ROC significantly, with greater baseline values associated with more rapid progression [22,23]. Baseline values of global BMO-MRW and global RNFL were 202.12 ± 44.47 µm and 79.29 ± 12.65 µm, respectively, in our study. It was noticeable that baseline value of BMO-MRW was much greater than that of RNFL thickness, corresponding with previous studies [22,23]. Therefore, the ROC of BMO-MRW might be greater than that of RNFL thickness as demonstrated in our study and previous studies [22,23,24]. However, ROCs were not statistically significantly different between BMO-MRW and RNFL in our study of subjects with early NTG except for the superonasal sector. In our previous study, we have compared ROCs of BMO-MRW and RNFL in eyes showing optic disc hemorrhage (DH) that are prone to glaucomatous progression [24]. We found that the ROC of BMO-MRW was significantly greater than that of RNFL in eyes showing DH except for the nasal sector. ROCs of global BMO-MRW and RNFL were −3.507 ± 0.675 µm/yr and −1.404 ± 0.208 µm/yr, respectively, in subjects showing DH. It is noticeable that ROCs of both parameters in eyes showing DH were greater than those with early NTG in the present study. Since the ROC of early NTG is relatively smaller than that of eyes showing DH, it is assumed that the difference in ROC between these two parameters may not show statistical difference. Although there were no statistically significant differences between BMO-MRW and RNFL in patients with early NTG, ROC of BMO-MRW was consistently greater than that of RNFL, especially in inferotemporal and superotemporal sectors where initial glaucomatous change would usually occur [25,26]. ROCs of BMO-MRW and RNFL in the inferotemporal sector were −3.024 ± 0.882 µm/yr and −1.964 ± 0.363 µm/yr, respectively. ROC of BMO-MRW and RNFL in the superotemporal sector were −2.401 ± 0.775 µm/yr and −1.250 ± 0.309, respectively. Therefore, it is still more advantageous to detect early glaucomatous progressive change from BMO-MRW than from RNFL in early stage of NTG when standard visual field test may not show significant glaucomatous changes. Since ROC is important in the management of glaucoma, findings of our study are meaningful in the treatment of early NTG. Even when RNFL does not show substantial glaucomatous deterioration, BMO-MRW may show a certain or significant amount of reduction in early NTG. In such case, clinicians should consider enhancing the intensity of treatment or shorten the interval of follow-up.

Bowd et al. [23] have reported that sites of glaucomatous changes are principally temporal and inferior sectors for BMO-MRW in all groups of diagnosis. Inferotemporal and superotemporal sectors are areas where early glaucomatous changes most frequently occur [25,26]. In the current study, only superonasal sector showed statistical difference in ROC between BMO-MRW and RNFL, with the ROC of BMO-MRW being greater than that of RNFL. We are not sure why this superonasal sector, which is not a main sector of initial glaucomatous injury shows such finding. Sectoral analysis of our study has taken account FoBMO axis to adjust individual cyclotorsion of eyes, which enables better analysis according to each Garway-Heath sector. In this regard, sectoral analysis has been performed precisely. However, other factors might have affected this result. Nonetheless, changes of the superonasal sector were better reflected by BMO-MRW than by RNFL.

The ROC of RNFL was slightly greater than that of BMO-MRW only in the temporal sector. This was an opposite finding from all the other sectors and global region. However, the difference between the two parameters was very small and the actual ROC was also very small. It was −0.785 ± 0.173 µm/yr for RNFL and −0.586 ± 0.444 µm/yr for BMO-MRW. The temporal sector showed the least ROC among all sectors and global region. Considering that the temporal sector was a less involved sector of initial glaucomatous damage and that temporal ROCs of BMO-MRW and RNFL were small with very small difference between the two parameters, our results still indicate the main trend of ROC of BMO-MRW being greater than that of RNFL. Therefore, BMO-MRW can provide more easily detectable reduction rate of glaucomatous deterioration than RNFL in early stage of NTG.

BMO is the outer border of the neuroretinal tissue at the optic disc. Axons of retinal ganglion cells run thorough BMO [27,28]. BMO-MRW can precisely reflect the amount of neuroretinal tissue at the optic disc [7,8,19,29]. BMO-MRW is measured directly at the optic disc while RNFL is measured at the peripapillary area at the scan circle of usually 3.5 mm. If there is any alteration (for example, large peripapillary atrophy or posterior vitreous detachment) at the scan circle, measurement of conventional RNFL thickness may not be correct. It may also be variable at each measurement. The abundant amount of neural tissue at the optic disc enables much reliable measurement each time compared to relatively thin RNFL. Therefore, it is more convenient to see changes over time with BMO-MRW than with RNFL. Simply because the actual value of BMO-MRW is greater than that of RNFL, it does not necessarily indicate that values of BMO-MRW are more variable than values of RNFL. Previous studies have revealed that BMO-MRW offers excellent intra- and interobserver reproducibility and intraday repeatability [30,31,32]. The thicker the measurement tissue is, the more there is left to measure over time. Therefore, it may be more beneficial to use BMO-MRW to see the rate of change in glaucoma than to use RNFL.

The present study has several limitations. One potential limitation was the nature of its retrospective research. We included only those subjects who had acquired more than five times of both BMO-MRW and RNFL image scans with reliable quality. The effect of such inclusion of subjects on our study results is unknown. Second, the present study was performed at a tertiary university hospital of the province with a hospital-based design. It was not a population-based study. Therefore, subjects included in this study might not represent the whole population of NTG patients of Asians. Third, the sample size of the current study should also be regarded, although a longitudinal design may have limited extensive inclusion. In addition, the follow-up period was not so long, although it was 20.99 ± 6.99 months with a mean number of 7.54 ± 2.12 for OCT tests. Nevertheless, including 115 subjects with a follow-up of almost two years may be sufficient to demonstrate the trend of ROC in a single disease of NTG. A further longitudinal study with a large number of subjects, a multicenter design, and a long-term follow-up is required.

In conclusion, we found that the ROC of BMO-MRW was consistently greater than that of RNFL in global region, especially in inferotemporal and superotemporal sectors of patients with early NTG, although their differences were not statistically significant. Considering that initial glaucomatous damage would occur mainly at inferotemporal and superotemporal sectors, these findings are clinically important in the management of early NTG. To the best of our knowledge, longitudinal ROC of BMO-MRW as a new parameter compared to RNFL has not been reported yet, particularly in a single ethnic group of Asians (Koreans). Since BMO-MRW can show more visible reduction rate of glaucomatous change than RNFL, BMO-MRW may be more advantageous to detect glaucomatous deterioration than RNFL in early NTG when standard automated perimetry may not show significant change. A further population-based study with a large number of subjects is required to have conclusive answers.

## Figures and Tables

**Table 1:** Baseline characteristics of included subjects of Early Normal Tension Glaucoma.

Characteristics	Values
Number of subjects	115 eyes (115 subjects)
Mean Age (year)	56.08 ± 10.94
Female gender (%)	53/115 (46.09%)
Family history of glaucoma (%)	11/115 (9.57%)
Disc hemorrhage (%)	35/115 (30.43%)
Mean follow-up period (months)	20.99 ± 6.99
Mean number of OCT tests	7.54 ± 2.12
Quality score of RNFL	29.44 ± 3.53
Quality score of BMO-MRW	32.38 ± 3.13
FoBMO angle (°)	−6.16 ± 3.17
NTG	115/115 (100%)
Spherical equivalent (D)	−1.69 ± 2.81
CCT (µm)	540.13 ± 36.76
Baseline IOP (mmHg)	14.72 ± 2.70
VFI (%)	93.30 ± 6.40
MD (dB)	−2.73 ± 2.26
PSD (dB)	4.75 ± 3.02

OCT = optical coherence tomography; NTG = normal tension glaucoma; CCT = central corneal thickness; D = diopters; IOP = intraocular pressure; VFI = visual field index; MD = mean deviation; PSD = pattern standard deviation.

**Table 2:** Progression rate of BMO-MRW and RNFL per year in Early Normal Tension Glaucoma.

Outcome	Baseline Value/ Progression Rate	Standard Error	95% CI		*p*-Value
			Lower	Upper	
**BMO Area**	2.29 *	0.54			
	0.00462 †	0.004336	−0.00389	0.01313	0.287
**BMO-MRW G**	202.12 *	44.47			
	−2.063 †	0.649	−3.338	−0.788	0.0016
**RNFL G**	79.29 *	12.65			
	−0.956 †	0.160	−1.270	−0.643	<0.0001
**BMO-MRW T**	158.60 *	38.28			
	−0.586 †	0.444	−1.458	0.287	0.1878
**RNFL T**	65.10 *	13.84			
	−0.785 †	0.173	−1.124	−0.447	<0.0001
**BMO-MRW TS**	202.81 *	59.34			
	−2.401 †	0.775	−3.923	−0.879	0.0020
**RNFL TS**	108.24 *	29.87			
	−1.250 †	0.309	−1.857	−0.644	<0.0001
**BMO-MRW TI**	180.54 *	64.17			
	−3.024 †	0.882	−4.756	−1.292	0.0006
**RNFL TI**	92.17 *	32.91			
	−1.964 †	0.363	−2.678	−1.251	<0.0001
**BMO-MRW N**	224.29 *	63.35			
	−1.844 †	1.100	−4.004	0.317	0.0943
**RNFL N**	65.37 *	15.96			
	−0.502 †	0.222	−0.937	−0.067	0.0237
**BMO-MRW NS**	231.50 *	70.13			
	−4.077 †	0.826	−5.699	−2.455	<0.0001
**RNFL NS**	95.78 *	30.30			
	−0.974 †	0.364	−1.689	−0.259	0.0076
**BMO-MRW NI**	230.57 *	59.59			
	−2.340 †	1.066	−4.432	−0.248	0.0284
**RNFL NI**	89.54 *	19.28			
	−0.882 †	0.251	−1.375	−0.390	0.0005

BMO-MRW = Bruch membrane opening minimum rim width; RNFL = retinal nerve fiber layer; G = global; T = temporal; TS = superotemporal; NS = superonasal; N = nasal; NI = inferonasal; TI = inferotemporal. *p*-Value by generalized linear mixed model including random intercept for subjects after adjusting for age, sex, and BMO area. Coefficient by time is calculated progression rate per year (µm/yr). * Baseline value (um), † coefficient as progression rate (µm/yr).

**Table 3:** Comparison of progression rate between BMO-MRW and RNFL in each sector.

Compare Coefficients by Sector		
Sector	Z-Score	*p*-Value
**G**	−1.6547	0.0980
**TMP**	0.4185	0.6756
**TS**	−1.3788	0.1680
**TI**	−1.1109	0.2666
**NAS**	−1.1952	0.2320
**NS**	−3.4375	**0.0006**
**NI**	−1.3317	0.1830

BMO-MRW = Bruch membrane opening minimum rim width; RNFL = retinal nerve fiber layer; G = global; T = temporal; TS = superotemporal; NS = superonasal; N = nasal; NI = inferonasal; TI = inferotemporal. *p*-Value by *t*-test. **Bold font** indicates significant *p* values (*p* < 0.05).

**Table 4:** Comparison of progression rate between BMO-MRW and RNFL in percent reduction.

Compare Coefficients by Sector (Percent Reduction)				
Sector	Percent Coefficient, BMO-MRW	Percent Coefficient, RNFL	Z-Score	*p*-Value
**G**	−1.6394 ± 0.5161	−1.2006 ± 0.2005	−0.7926	0.4280
**T**	−0.2576 ± 0.1954	−1.0468 ± 0.2300	2.6150	0.0089
**TS**	−1.7785 ± 0.5743	−1.1964 ± 0.2954	−0.9013	0.3674
**TI**	−1.6193 ± 0.4724	−2.6363 ± 0.4876	1.4979	0.1342
**N**	−3.8762 ± 2.3132	−0.7494 ± 0.3307	−1.3381	0.1809
**NS**	−2.3742 ± 0.4810	−1.0316 ± 0.3856	−2.1780	**0.0294**
**NI**	−2.4171 ± 1.1006	−0.9464 ± 0.2690	−1.2980	0.1943

BMO-MRW = Bruch membrane opening minimum rim width; RNFL = retinal nerve fiber layer; G = global; T = temporal; TS = superotemporal; NS = superonasal; N = nasal; NI = inferonasal; TI = inferotemporal. *p*-Value by *t*-test. **Bold font** indicates significant *p*-values (*p* < 0.05).

**Table 5:** Progression rate of BMO-MRW and RNFL after standardization.

Outcome		Standardized Coefficient	95% CI	
			Lower	Upper
**BMO-MRW G**	Intercept (µm)	0.922	−0.400	2.242
	Time (µm/yr)	−0.047	−0.077	−0.018
**RNFL G**	Intercept (µm)	0.050	−1.029	1.130
	Time (µm/yr)	−0.079	−0.105	−0.053
**BMO-MRW T**	Intercept (µm)	2.137	1.228	3.046
	Time (µm/yr)	−0.016	−0.039	0.008
**RNFL T**	Intercept (µm)	0.782	−0.244	1.809
	Time (µm/yr)	−0.056	−0.080	−0.032
**BMO-MRW TS**	Intercept (µm)	0.605	−0.560	1.769
	Time (µm/yr)	−0.041	−0.067	−0.015
**RNFL TS**	Intercept (µm)	−0.101	−1.089	0.886
	Time (µm/yr)	−0.041	−0.060	−0.021
**BMO-MRW TI**	Intercept (µm)	1.011	−0.008	2.029
	Time (µm/yr)	−0.047	−0.074	−0.020
**RNFL TI**	Intercept (µm)	−0.442	−1.459	0.576
	Time (µm/yr)	−0.060	−0.082	−0.038
**BMO-MRW N**	Intercept (µm)	0.484	−0.959	1.927
	Time (µm/yr)	−0.029	−0.064	0.005
**RNFL N**	Intercept (µm)	0.046	−1.077	1.168
	Time (µm/yr)	−0.038	−0.070	−0.005
**BMO-MRW NS**	Intercept (µm)	0.137	−0.950	1.223
	Time (µm/yr)	−0.060	−0.084	−0.036
**RNFL NS**	Intercept (µm)	−0.159	−1.207	0.890
	Time (µm/yr)	−0.034	−0.058	−0.009
**BMO-MRW NI**	Intercept (µm)	−0.034	−1.245	1.177
	Time (µm/yr)	−0.037	−0.071	−0.004
**RNFL NI**	Intercept (µm)	0.197	−0.850	1.244
	Time (µm/yr)	−0.045	−0.071	−0.020

Generalized linear mixed model including random intercept for subjects after standardization. Coefficient by time is calculated progression rate per year (µm/yr) after standardization. BMO-MRW = Bruch membrane opening minimum rim width; RNFL = retinal nerve fiber layer; G = global; T = temporal; TS = superotemporal; NS = superonasal; N = nasal; NI = inferonasal; TI = inferotemporal.
